# Microarray analysis of DNA damage repair gene expression profiles in cervical cancer cells radioresistant to ^252^Cf neutron and X-rays

**DOI:** 10.1186/1471-2407-10-71

**Published:** 2010-02-25

**Authors:** Yi Qing, Xue-Qin Yang, Zhao-Yang Zhong, Xin Lei, Jia-Yin Xie, Meng-Xia Li, De-Bing Xiang, Zeng-Peng Li, Zhen-Zhou Yang, Ge Wang, Dong Wang

**Affiliations:** 1 Cancer Center, Daping Hospital and Research Institute of Surgery, Third Military Medical University, Chongqing, PR China; 2 Department of Pathology, Daping Hospital and Research Institute of Surgery, Third Military Medical University, Chongqing, PR China

## Abstract

**Background:**

The aim of the study was to obtain stable radioresistant sub-lines from the human cervical cancer cell line HeLa by prolonged exposure to ^252^Cf neutron and X-rays. Radioresistance mechanisms were investigated in the resulting cells using microarray analysis of DNA damage repair genes.

**Methods:**

HeLa cells were treated with fractionated ^252^Cf neutron and X-rays, with a cumulative dose of 75 Gy each, over 8 months, yielding the sub-lines HeLaNR and HeLaXR. Radioresistant characteristics were detected by clone formation assay, ultrastructural observations, cell doubling time, cell cycle distribution, and apoptosis assay. Gene expression patterns of the radioresistant sub-lines were studied through microarray analysis and verified by Western blotting and real-time PCR.

**Results:**

The radioresistant sub-lines HeLaNR and HeLaXR were more radioresisitant to ^252^Cf neutron and X-rays than parental HeLa cells by detecting their radioresistant characteristics, respectively. Compared to HeLa cells, the expression of 24 genes was significantly altered by at least 2-fold in HeLaNR cells. Of these, 19 genes were up-regulated and 5 down-regulated. In HeLaXR cells, 41 genes were significantly altered by at least 2-fold; 38 genes were up-regulated and 3 down-regulated.

**Conclusions:**

Chronic exposure of cells to ionizing radiation induces adaptive responses that enhance tolerance of ionizing radiation and allow investigations of cellular radioresistance mechanisms. The insights gained into the molecular mechanisms activated by these "radioresistance" genes will lead to new therapeutic targets for cervical cancer.

## Background

Cervical cancer is a worldwide disease, with a high incidence and mortality [1] especially in developing countries. Approximately 500,000 cases of cervical cancer are diagnosed annually, with mortality around 40% [2]. Radiotherapy is particularly effective for patients with cervical cancers at an advanced stage or that cannot be cured surgically. Low LET radiation (e.g., gamma rays, X-rays) is generally used to treat cervical cancers, but its ability to cure local disease decreases with increasing tumor size, because the doses required to treat large tumors exceed the toxicity limits of normal tissues [3]. High LET radiation such as neutron rays is associated with a low rate of repair of potentially lethal DNA damage, allowing better local control of the tumor and thus less chance of recurrence. Theoretically, high LET radiation has several advantages over low LET radiation: (a) more damage to hypoxic cells; (b) decreased repair of IR-induced damage; and (c) effectiveness at all stages of the cell cycle [4]. The 5-year overall survival rate of cervical cancer patients who received ^252^Cf neutron ray radiotherapy was reported to be 76.8% at stage II and 70.9% at stage III [5]. Another study showed that the 10-year survival rate was better in cervical cancer patients treated with ^252^Cf neutron rays than in those treated with gamma radiation (69.1% vs. 50.9%, respectively) [6]. However, despite the improved efficacy of ^252^Cf neutron rays in the therapy of cervical cancer, tumors recurrence has been reported.

A substantial body of evidence has implicated DNA as the primary target in lethal ionizing radiation (IR) [7]. In many human tumor lines, radiosensitivity correlated with DNA damage induction and repair [8-10]. The activity of the DNA damage repair pathway is one of the most important factors leading to radioresistance in tumors, including cervical cancer. This pathway as well as others contributing to radioresistance in cervical cancer can be studied by cDNA microarray analyses of gene expression. Thus, in the present work, long-term ^252^Cf neutron ray and X-ray irradiation of HeLa cells was used to generate two radioresistant cell sub-lines, HeLaNR and HeLaXR, which provided a model system for studying the radioresistance mechanisms of cervical cancer cells. Insights into the mechanisms of resistance will contribute to improvements in cancer treatment.

## Methods

### Cell lines

The HeLa cell line was obtained from the American Type Culture Collection and cultured in DMEM-high glucose medium (GIBCO) supplemented with 10% newborn calf serum (Biochrom AG), penicillin (100 U/ml), and streptomycin (100 μg/ml) (Sigma). Cells were incubated in 5% CO_2 _at 37°C and passaged 2-3 times weekly. ^252^Cf neutron ray (HeLaNR) and X-ray (HeLaXR) resistant sub-lines were generated by continuous sublethal irradiation for 8 months with ^252^Cf neutron and X-rays, respectively, with a total dose of 75 Gy each. The parental cell line (HeLa) and the radioresistant sub-lines (HeLaNR and HeLaXR) were maintained under the same conditions. To control for acute effects of IR, the radioresistant sub-lines were cultured for over 2 months after the last irradiation before being used in the analyses.

### Irradiation

For X-ray treatment, cells were cultured in 25-cm^2 ^flasks until they reached 75% confluence and then irradiated at 200 cGy/min, at room temperature, with a Precise linear accelerator (Elekta) operating at 8 MV. For ^252^Cf neutron ray treatment, trypsinized cells were harvested in a tube and then irradiated with a neutron emission rate of 2.3 × 10^6^/s/μg, a γ emission rate of 1.3 × 10^7^/s/μg, and a dose rate in air of 23.4 mSv/h•mg at 1 cm from the source.

### Colony formation assay

HeLa, HeLaNR, and HeLaXR cells were treated with 0, 2, 4, 6, 8, and 10 Gy of ^252^Cf neutron or X-ray radiation. After 2 weeks, clones were fixed with methanol and stained with a 2% Giemsa solution (Merck) for 10 min. Stained clones that had more than 50 cells were counted and cloning efficiency calculated as: cloning efficiency = (clone number/total cell number)*100%. The cell survival fraction (SF) was determined. A cell survival curve was drafted using Microsoft Excel 2003 software and the single-hit multi-target model SF = 1- (1-e^-D/D0^)^N^, where SF is the survival fraction; D, the radiation dose; D_0_, the mean death dose; and N, the extrapolated number. D_q_, the standard threshold dose, was determined from logN = D_q_/D_0_; and SF2, the fraction surviving at 2 Gy, from the cell survival curve.

### Transmission electron microscopy (TEM)

HeLa, HeLaNR, and HeLaXR cells were harvested in exponential growth phase and centrifuged at 1500 rpm for 10 min at 4°C. Cell pellets were fixed in a 2.5% glutaraldehyde solution. Fixed cells were washed twice with PBS, dehydrated three times sequentially in a graded series of acetone solutions, and then incubated overnight in a 1:1 volume ratio of 100% alcohol and embedding resin. Resin-embedded cells were placed in capsules and then transferred to a Pelco UV-2 Cryo Chamber, where they were polymerized with UV irradiation at 4°C for 48 h. Ultrathin sections were prepared and then stained with uranyl acetate and lead citrate. Cellular uptake of nanoparticles was evaluated by TEM (TECNAI10, Philips).

### Cell doubling time

HeLa, HeLaNR, and HeLaXR cells were trypsinized in exponential growth phase. Equal numbers of cells (about 1 × 10^4^) were plated in 6-well culture plates (day 0) and cultured in 5% CO_2 _at 37°C. Viable cells were determined by the trypan blue exclusion test and counted in triplicate on days 1, 2, 3, 4, 5, and 6.

### Cell cycle analysis

HeLa, HeLaNR, and HeLaXR cells were treated with 0, 4, and 16 Gy of ^252^Cf neutron ray and X-ray radiation. At 24 h post-irradiation, the cells were harvested, fixed with 70% ethanol, and then stored at 4°C. Cell cycle analysis was carried out by flow cytometry (FACSCalibur, Becton Dickinson) at 488 nm.

### Apoptosis analysis

HeLa, HeLaNR and HeLaXR cells were treated with 0, 4 and 16 Gy of ^252^Cf neutron ray and X-ray radiation. At 48 h post-irradiation, the cells were harvested and then stained with annexin V-FITC and PI (Invitrogen). Cell apoptosis analysis was carried out by flow cytometry.

### RNA preparation and microarray analysis

Total RNA was isolated from HeLa, HeLaNR, and HeLaXR cells with TRIzol^® ^(Invitrogen) according to the manufacturer's instructions. SuperArray (Oligo GEArray^® ^Human DNA Damage Signaling Pathway Microarray) was used following the manufacturer's protocol to compare the gene expression profiles of the three cell lines, and the results interpreted with the GEArray Expression Analysis Suite. SuperArray contains 113 functionally well-characterized genes associated with the ATR/ATM signaling network, including transcriptional targets of DNA damage response (cell cycle arrest, apoptosis, and DNA repair). Controls consist of four normalizable genes (glyceraldehyde-3-phosphate dehydrogenase, beta-2-microglobulin, heat-shock protein 90-α, and β-actin) and a blank. The experiment was performed more than three times using different RNA preparations. The microarray data had been deposited in GEO database of NCBI. The Series number was GSE19526 http://www.ncbi.nlm.nih.gov/geo/query/acc.cgi?acc=GSE19526 and the Platform number was GPL9811 http://www.ncbi.nlm.nih.gov/geo/query/acc.cgi?acc=GPL9811.

### Western blotting

Total protein extracts were prepared from HeLa, HeLaNR, and HeLaXR cells. The proteins (20 μg/sample) were resolved by electrophoresis on 12% SDS-polyacrylamide gels, transferred to polyvinylidene difluoride membranes, and blocked in TBST [50 mM Tris-HCl, pH 7.5, 150 mM NaCl and 0.1% (v/v) Tween 20] containing 5% (w/v) nonfat dry milk. The membrane was incubated with a 1:2000 dilution of GADD45α (mouse monoclonal antibody, Abnova) and a 1:1000 dilution of BTG2 (goat polyclonal IgG, Santa Cruz) primary antibody overnight at 4°C, washed three times in TBST, and then incubated with a 1:2000 dilution of horseradish-peroxidase-labeled secondary antibody (Pierce) for 1 hour at 37°C. The membrane was then reacted with chemiluminescent reagents (Pierce) and exposed onto film (Kodak). Bands intensities were analyzed using the Gel Doc 2000 apparatus and software (Quantity One, Bio-Rad). Protein loading was normalized with β-actin (Sigma).

### Real-time PCR

Total RNA was isolated from HeLa, HeLaNR, and HeLaXR cells. Reverse transcription was performed with AMV reverse transcriptase (TaKaRa) and oligodT18 primers (TaKaRa). Real-time PCR was carried out using SYBR Green I (Molecular Probes) and the iQCycler thermocycler (Bio-Rad). Relative expression of the GADD45α and BTG2 genes was measured by real-time quantitative RT-PCR using β-actin as internal control for possible contamination of genomic DNA and for normalization of variation in the amounts of PCR product loaded. Primer sequences were: 5'-AGTCAGCGCACGATCACTGTC-3' (forward) and 5'-GACGCGCAGGATGTTGATGTC-3' (reverse) for GADD45α, which generated a 183-bp fragment; 5'-ACATGAGCCACGGGAAGGGAAC-3' (forward) and 5'-ATGCGAATGCAGCGGTAGCC-3' (reverse) for BTG2, which generated a 211-bp fragment. Real-time PCR was carried out as follows: initial denaturation for 5 min at 94°C and 42 PCR cycles consisting of 15 s at 94°C, 15 s at 58°C, and 18 s at 72°C. Standards and cDNA samples were amplified in triplicate in the same reaction plate in at least three independent experiments.

### Statistical analysis

Quantitative data were obtained from three independent experiments and expressed as mean ± SD. Statistical difference between two groups was determined using Student's *t *test. *P *values were-two sided; *P *< 0.05 was considered as statistically significant.

## Results

### Radiosensitivity

**Figure 1:**
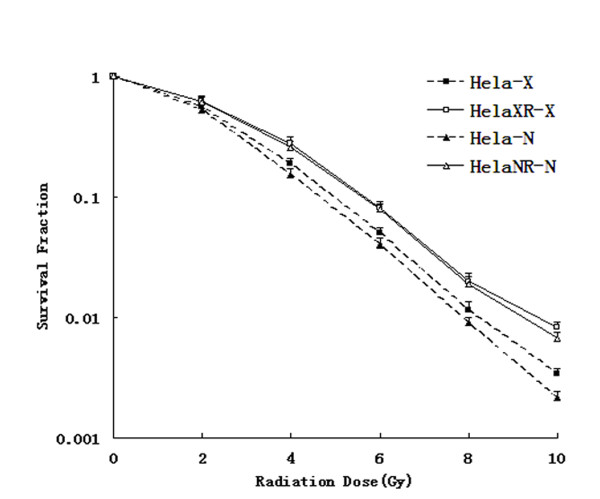
**Survival fraction curves of HeLa, HeLaNR and HeLaXR cells**. The radiation survival fraction curve derived from colony formation assays of HeLa, HeLaNR, and HeLaXR cells after different doses of ^252^Cf neutron rays and X-rays. HeLaNR and HeLaXR cells are more radioresistant to IR than parental cells.

Radiation-survival curves, generated according to the above-described methods, were derived from colony formation assays of the three cell lines after different doses of ^252^Cf neutron and X-rays (Figure 1). These curves were then used to determine D_0_, D_q_, and SF2 (Table 1). Under equal doses of ^252^Cf neutron ray and X-ray irradiation, the D_0_, D_q_, and SF2 values of the HeLaNR and HeLaXR cells were higher than those of the HeLa cells, indicating that the sub-lines were more radioresistant than the parent cells.

**Table 1:** D_0_, D_q_, and SF2 values in HeLa, HeLaNR, and HeLaXR cells

Group	**D**_**0 **_**(Gy)**	**D**_**q **_**(Gy)**	**SF**_**2**_
HeLa-N	1.45	0.59	0.52
HeLaNR-N	1.84	0.67	0.61
HeLa-X	1.58	0.61	0.55
HeLaXR-X	1.88	0.7	0.63

### Ultrastructure

**Figure 2:**
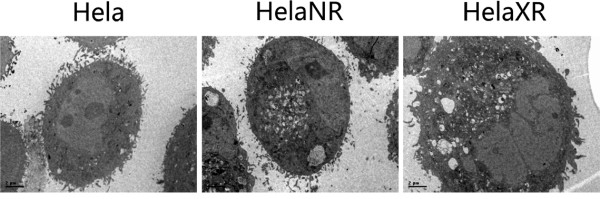
**Ultrastructural features of HeLa, HeLaNR, and HeLaXR cells examined by TEM (×3,700)**. Microvilli are present on the HeLa cell surface and the cytoplasm contains abundant mitochondria and ribosome. In the radioresistant sub-lines, mitochondrial swelling, vacuolization, dilatation of the endoplasmic reticulum, and myelin figures are seen.

The ultrastructure of HeLa, HeLaNR, and HeLaXR cells was examined by TEM (Figure 2). In HeLa cells, microvilli were present on the surface and the cytoplasm contained abundant mitochondria and ribosomes. In the radioresistant sub-lines, swelling of mitochondria, vacuolization, dilatation of the endoplasmic reticulum, and myelin figures were observed. Thus, in the HeLaNR and HeLaXR sub-lines, the ultrastructural changes induced by long-term irradiation persisted even > 2 months after the last radiation treatment.

### Cell proliferation

**Figure 3:**
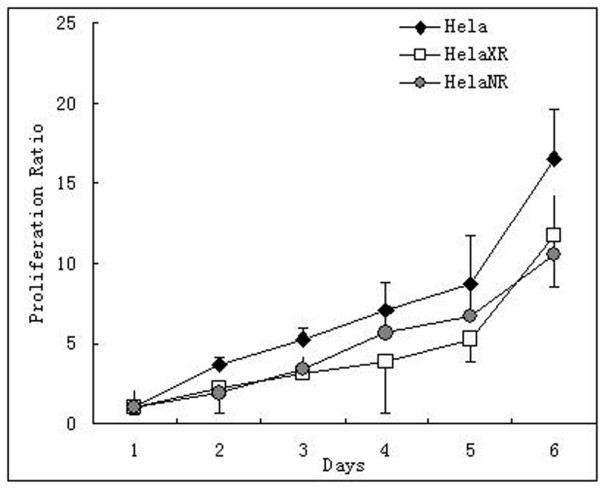
**Cell growth curves of HeLa, HeLaNR and HeLaXR cells**. The cell doubling time of HeLaNR and HeLaXR cells is 33.12 ± 3.67 h and 36.94 ± 3.16 h, respectively, is longer than that of the parent HeLa cells (28.62 ± 2.77 h).

Cell doubling time, *t*_d_, was estimated by the following formula:

where *X*_1 _and *X*_2 _are the number of cultured cells at the current (*t*_2_) and previous (*t*_1_) measurements. As shown in Figure 3, the cell doubling times of the HeLaNR and HeLaXR cells (33.12 ± 3.67 h, and 36.94 ± 3.16 h, respectively) were longer than those of the parent HeLa cells (28.62 ± 2.77 h).

### Cell cycle distribution

**Figure 4:**
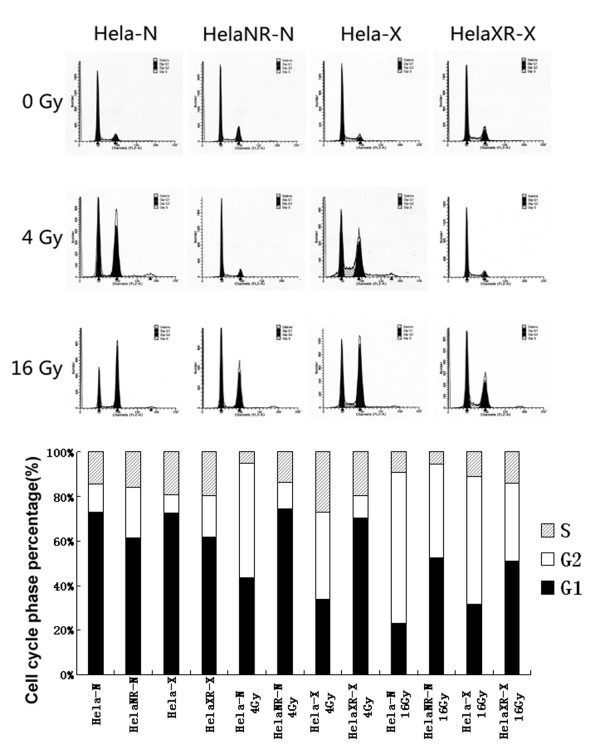
**Cell cycle distribution in the parent HeLa cells and the two radioresistant sub-lines 24 h after irradiation**. Exposure of HeLa cells to a radiation dose of 4 Gy significantly increased the proportion of cells in the G2 phase of the cell cycle and decreased the proportion of cells in G1. The degree of G2 arrest was more apparent after irradiation with 16 Gy. The cell cycle distribution pattern of the radioresistant sub-lines also changed but without an increase in G2 cells after irradiation with 4 Gy. Increasing the irradiation doses to 16 Gy induced G2 arrest but the proportion of arrested cells was much lower than in the HeLa cell population.

The cell cycle distribution of HeLa, HeLaNR, and HeLaXR cells 24 h after ^252^Cf neutron ray and X-ray irradiation was determined by flow cytometry (Figure 4). In HeLa cells, exposure to a radiation dose of 4 Gy significantly increased the proportion of cells in G2 and decreased the proportion of cells in G1. The proportion of G2-arrested HeLa cells was even greater following 16-Gy irradiation. In the HeLaNR and HeLaXR sub-lines, however, the proportion of cells in G2 did not increase after irradiation with 4 Gy. At 16 Gy, cells of both sub-lines became arrested in G2 but the proportion was much lower than in HeLa cells. Thus, cells of the radioresistant sub-lines probably arrested in the G1 phase of the cell cycle.

### Cell apoptosis

**Figure 5:**
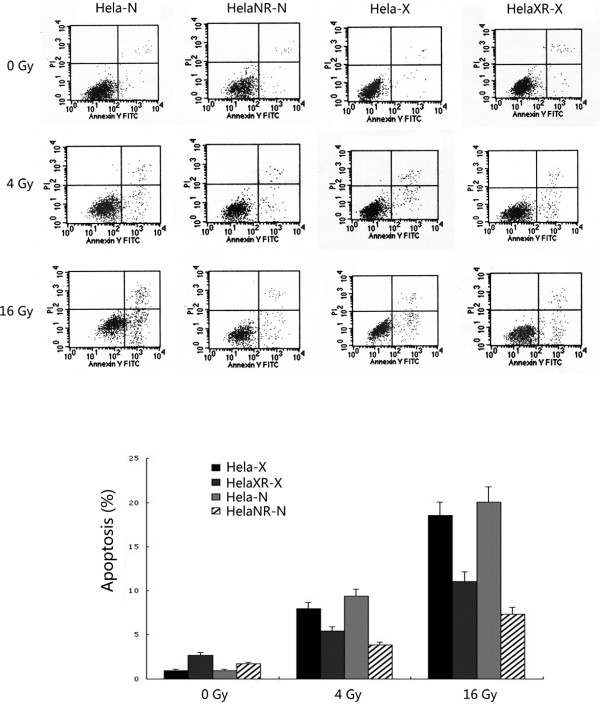
**Apoptosis assay in three cells after irradiation at 48 h. Apoptosis assay of HeLa cells and the radioresistant two sub-lines 48 h after irradiation**. At 0 Gy, the apoptosis rate was 0.93-2.71%for all three cell lines. The difference in the apoptosis rate between HeLa cells vs. HeLaXR and HeLaNR cells significantly increased with higher radiation doses (4 Gy: 9.32 *vs *3.84, 7.94 *vs *5.43; 16 Gy: 22.47 *vs *7.28, 20.03 *vs *11.1, *p *< 0.05).

The level of apoptosis was analyzed by flow cytometry 48 h after 0, 4, and 16 Gy ^252^Cf neutron ray and X-ray irradiation (Figure 5). At 0 Gy, the apoptosis rate was 0.93-2.71% for all three cell lines. At 4 and 16 Gy, the apoptosis rate of HeLa cells was much higher than that of the radioresisitant sub-lines (4 Gy: 9.32 *vs *3.84, 7.94 *vs *5.43; 16 Gy: 22.47 *vs *7.28, 20.03 *vs *11.1, *p *< 0.05). Thus, the sub-lines HeLaXR and HeLaNR were more radioresistant than the parental HeLa cell line.

### Gene expression

Using stringent criteria for array analysis (≥2-fold change in expression), we identified 113 genes related to DNA damage signaling pathways that were differentially expressed in the parental HeLa cells compared to radioresistant HeLaXR and HeLaNR cells (Table 2). Of the 24 genes significantly altered by at least 2-fold in HeLaNR cells, 19 were up-regulated and 5 down-regulated. Likewise, of the 41 genes significantly altered by at least 2-fold in HeLaXR cells, 38 were up-regulated and 3 down-regulated. For the two radioresistant sub-lines, the similar overall trend in gene-expression changes indicated that long-term exposure to ^252^Cf neutron and X-rays had resulted in a similar induction of genes involved in DNA damage signaling pathways. But genes encoding homologous recombination, nonhomologous end-joining, mismatch repair, and cell cycle arrest functions were more highly expressed in cells resistant to X-rays than in cells resistant to ^252^Cf neutron rays.

**Table 2:** Gene expression changes in HeLaNR and HeLaXR cell lines (≥2-fold)

GeneBank	Symbol	Gene Function	Regulation Mode	
			
			HeLaNR*	HeLaXR*
NM_000051	ATM	Other genes involved in DNA repair		up
NM_006763	BTG2	Other genes involved in DNA repair	up	
NM_001279	CIDEA	Apoptosis genes	up	up
NM_014430	CIDEB	Apoptosis genes	up	up
NM_000082	CKN1	Nucleotide excision repair	up	
NM_001923	DDB1	Nucleotide excision repair	up	up
NM_007068	DMC1	Nucleotide excision repair		up
NM_005236	ERCC4	Nucleotide excision repair	up	
NM_006705	GADD45G	Apoptosis genes	up	
NM_001515	GTF2H2	Nucleotide excision repair	up	
NM_016426	GTSE1	Cell cycle arrest		up
NM_004507	HUS1	Cell cycle arrest		up
NM_054111	IHPK3	Apoptosis genes	up	
NM_001567	INPPL1	Other genes involved in DNA repair	up	up
NM_033276	KUB3	Nonhomologous end-joining	up	up
NM_000234	LIG1	Nucleotide excision repair		up
NM_002758	MAP2K6	Cell cycle arrest		up
NM_002969	MAPK12	Cell cycle arrest	up	up
NM_005590	MRE11A	Homologous recombination		up
NM_018177	N4BP2	Other genes involved in DNA repair		up
NM_002528	NTHL1	Base excision repair	up	up
NM_020418	PCBP4	Apoptosis genes		up
D38500	PMS2L4	Mismatch repair		up
NM_005395	PMS2L9	Mismatch repair		up
NM_007254	PNKP	Base excision repair	up	up
NM_014330	PPP1R15A	Apoptosis genes		up
NM_006904	PRKDC	Nonhomologous end-joining		up
NM_002873	RAD17	Cell cycle arrest		up
NM_006265	RAD21	Apoptosis genes	up	
NM_005053	RAD23A	Nucleotide excision repair		up
NM_002875	RAD51	Homologous recombination		up
NM_133509	RAD51L1	Homologous recombination		up
NM_002878	RAD51L3	Homologous recombination		up
NM_002879	RAD52	Homologous recombination	up	up
NM_003579	RAD54L	Homologous recombination		up
NM_004584	RAD9A	Cell cycle arrest		up
NM_016316	REV1L	Other genes involved in DNA repair		up
NM_000546	TP53	Apoptosis genes		up
NM_016381	TREX1	Mismatch repair		up
NM_007205	TREX2	Other genes involved in DNA repair		up
NM_003362	UNG	Base excision repair	up	up
NM_021147	UNG2	Base excision repair	up	up
NM_005431	XRCC2	Homologous recombination		up
NM_005432	XRCC3	Homologous recombination	up	up
NM_003401	XRCC4	Nonhomologous end-joining		up
NM_001239	CCNH	Nucleotide excision repair		down
NM_004083	DDIT3	Cell cycle arrest	down	down
NM_001924	GADD45A	Apoptosis genes	down	down
NM_002311	LIG3	Base excision repair	down	
NM_000534	PMS1	Mismatch repair	down	
NM_002873	RAD17	Cell cycle arrest	down	

* compared with HeLa cells

### Western blotting and real-time PCR

**Figure 6:**
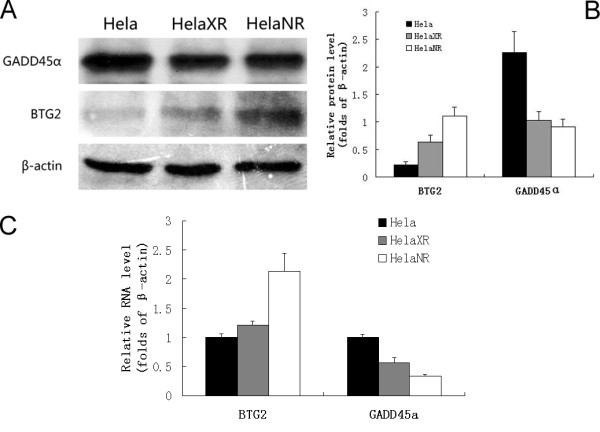
**BTG2 and GADD45α protein and mRNA expression in HeLa, HeLaNR and HeLaXR cells**. BTG2 protein expression is up-regulated in the sub-lines, especially HeLaNR. GADD45α protein expression is down-regulated in the sub-lines (A and B). Changes in BTG2 and GADD45α mRNA expression paralleled those in the protein levels (C).

Changes in gene expression detected in the microarrays were validated by Western blotting and real-time PCR. Based on the results shown in Figure 6, two genes, BTG2 and GADD45α, were chosen for further analysis and validation of the microarray data by Western blotting and real-time PCR. As shown by Western blotting, BTG2 protein expression was up-regulated in the resistant sub-lines, especially in HeLaNR cells, while the expression of GADD45α protein was down-regulated in both resistant sub-lines. PCR analysis showed that BTG2 and GADD45α mRNA expression paralleled that of the respective proteins.

## Discussion

Chronic exposure of cells to IR induced an adaptive response that resulted in enhanced tolerance to the subsequent cytotoxicity of IR [11-13]. In this study, HeLa cells were irradiated with fractionated ^252^Cf neutron and X-rays, yielding two radioresistant cell sub-lines. The mechanisms of this increased cellular radioresistance were investigated by comparing changes in the gene expression profiles of the parent HeLa cells with those of the HeLaNR and HeLaXR sub-lines.

Post-irradiation survival of the radioresistant sub-lines was significantly higher than that of the parental cells, with increased D_0 _and D_q_. This resistant phenotype appears to be stable because it did not change after continuous culture of the sub-lines for > 2 months in the absence of further irradiation. Similar findings were reported in the radioresistant human fibrosarcoma cell line HT1080R, obtained by long-term exposure to X-rays [14]. In *Escherichia coli*, radioresistant mutant bacterial strains were produced by daily X-ray irradiation [15]. Radiation resistance after long-term fractionated radiation has also been reported for breast [16], pancreatic [17], esophageal [18], and nasopharyngeal [19] cancer cells.

Tumor cells are heterogeneous with respect to their radioresponsiveness. The radioresistant phenotype is ascribed to several factors, including alterations in cell cycle checkpoints, slowed growth, and decreased apoptosis [20]. Here, the ultrastructure, proliferation, cell cycle distribution, and apoptosis rate of the radioresistant sub-lines were studied. As shown by TEM, HeLaNR and HeLaXR cells were ultrastructurally altered after long-term exposure to IR, even after > 2 months of culture after the last radiation dose. The cell doubling times of these sub-lines were longer than that of HeLa cells, consistent with the results of Russell *et al. *[11].

The cell cycle distribution pattern is strongly disturbed by irradiation; in turn, radiosensitivity depends on cell cycle position and cell cycle progression [21]. In a previous study, radiosensitive AT cells were shown to lack G1 arrest, accumulating instead at G2, while the G2 phase of radioresistant REC-derived cells was significantly prolonged [22]. We found that HeLaNR, HeLaXR, and HeLa cells differed in their cell cycle distribution, with HeLaNR and HeLaXR sub-lines most likely arrested in G1. This difference between the parent and the two sub-lines may reflect their different radiosensitivities. Likewise, IR-induced apoptosis was found to be better tolerated by the radioresistant HeLaNR and HeLaXR cells than by the parental HeLa cells. Previous studies provided evidence of the important role played by apoptosis in radiation-induced cell death and as a determinant of radiosensitivity [23,24].

DNA microarray technology is a powerful technique to detect the biological response of thousands of genes to external stimuli, including IR [25]. Gene expression profiling by DNA microarray has been applied to classify disease stages and predict treatment response to radiotherapy in cervical cancer [26-31]. In these previous studies, sets of genes associated with pathways such as apoptosis (e.g., bax, bcl-2), DNA damage repair (e.g., Ku80, GADD45, XRCC5), cell adhesion (e.g., ICAM-3), angiogenesis (e.g., HIF-1a), and tumor cell invasion (e.g., CTSL, CTSB) were identified.

Here, microarray analysis was used to identify the gene expression patterns of two radioresistant sub-lines derived from HeLa cells. Although many stress-responsive genes are inducible by IR [32], we found that the expression of only a small fraction of the radiation-inducible genes, such as those involved in cell cycle checkpoints, apoptosis, and DNA repair [33], was altered in our radioresistant sub-lines. For the two radioresistant sub-lines, the similar overall trend in gene-expression changes indicated that long-term exposure to ^252^Cf neutron and X-rays had resulted in a similar induction of genes involved in DNA damage signaling pathways. The number of genes that are involved in homologous recombination and nonhomologous end-joining, processes that result in altered gene expression, was higher in HeLaXR cells (8 and 3, respectively) than in HeLaNR cells (2 and 1, respectively). Furthermore, the number of cell cycle arrest and mismatch repair genes expressed was higher in HeLaXR cells (7 and 3, respectively) than in HeLaNR cells (3 and 1, respectively) whereas the number of base excision repair genes expressed in the two sub-lines was almost the same (4 in HeLaXR cells and 5 in HeLaNR cells). Hence, our results indicated that the expression of genes encoding homologous recombination, nonhomologous end-joining, mismatch repair, and cell cycle arrest functions was more closely related to X-ray radioresistance than to ^252^Cf neutron ray radioresistance.

BTG2 and GADD45α mRNA and protein expression levels were determined by real-time PCR and Western blot and the results confirmed the microarray data. BTG2 is an antiproliferative gene and one of the early growth response genes [34]. In a previous study, we investigated the expression of BTG2 and its role in hepatic cancer [35]. BTG2 overexpression inhibits G1-S progression through transcriptional regulation of cyclin D1 in the presence of pRB [36]. In NIH3T3 cells, forced expression of BTG2 provoked a marked inhibition of cell proliferation [34]. BTG2 was also identified as a differentiation and anti-apoptotic factor in neurogenesis [37]. In the present study, BTG2 mRNA and protein were found to be overexpressed in the two radioresistant sub-lines, suggesting that BTG2 plays an important role in resistance to IR-induced apoptosis and G1 arrest.

GADD45α is a downstream target of p53 and a member of a group of genes induced by DNA damaging agents and growth arrest signals. KLOPP et al [38] examined 12 cervical cancer patients with microarray and find that GADD45α is upregulated by radiation in NED (no evidence of disease) patients (1.13) and downregulated (0.96) in recurrent-disease patients (p = 0.36). Moreover, we find that the expression of GADD45α is downregulated in radioresitant sublines HeLaNR and HeLaXR. GADD45α is thought to be involved in cell cycle control at the G2-M checkpoint [39] and in the induction of cell death [40,41] following DNA damage. It may interact with MEKK4/MTK1 [40] and activates the JNK/p38 [41] signaling pathway, which induces apoptosis. Thus, low-level expression of GADD45α may in part explain the radioresistance of HeLaNR and HeLaXR cells to IR-induced apoptosis.

## Conclusions

In the present study, radioresistance mechanisms in cells chronically exposed to IR were investigated using DNA microarray analysis. Comprehensive expression profiles of genes involved in DNA damage signaling pathway were generated in HeLa and two radioresistant sub-lines in order to identify those genes whose expression correlated with radioresistance. Further investigations of these and other "radioresistance" genes will provide new insights in our understanding of the molecular mechanisms of radioresistance, potentially leading to new therapeutic targets for cervical cancer.

## Competing interests

The authors declare that they have no competing interests.

## Authors' contributions

YQ carried out most of the experimental work and contributed to draft the manuscript. XL and JYX contributed to the present work by accomplishing cell culture treatments and radiation of cell lines. MXL and DBX contributed to performed western blot and real-time PCR. ZYZ and ZZY performed cell cycle analysis and cell apoptosis. ZPL carried out TEM analysis. XQY contributed to microarray analysis. GW contributed to elaboration of data. DW participated and coordinated the study, compiled and finalized the manuscript. All authors read and approved the final manuscript.

## Pre-publication history

The pre-publication history for this paper can be accessed here:

http://www.biomedcentral.com/1471-2407/10/71/prepub
